# The Glasgow Microenvironment Score: an exemplar of contemporary biomarker evolution in colorectal cancer

**DOI:** 10.1002/2056-4538.12385

**Published:** 2024-06-09

**Authors:** Katrina Knight, Christopher Bigley, Kathryn Pennel, Jennifer Hay, Noori Maka, Donald McMillan, James Park, Campbell Roxburgh, Joanne Edwards

**Affiliations:** ^1^ Academic Unit of Surgery, Glasgow Royal Infirmary, School of Medicine, Dentistry and Nursing University of Glasgow Glasgow UK; ^2^ School of Cancer Sciences University of Glasgow Glasgow UK; ^3^ Glasgow Tissue Research Facility Queen Elizabeth University Hospital Glasgow UK; ^4^ Department of Pathology Queen Elizabeth University Hospital Glasgow UK; ^5^ Department of Surgery Queen Elizabeth University Hospital Glasgow UK

**Keywords:** colonic cancer, rectal cancer, biomarkers, tumour microenvironment, inflammation, stromal invasion, histopathology, subtypes, prognosis, microenvironment score

## Abstract

Colorectal cancer remains a leading cause of mortality worldwide. Significant variation in response to treatment and survival is evident among patients with similar stage disease. Molecular profiling has highlighted the heterogeneity of colorectal cancer but has had limited impact in daily clinical practice. Biomarkers with robust prognostic and therapeutic relevance are urgently required. Ideally, biomarkers would be derived from H&E sections used for routine pathological staging, have reliable sensitivity and specificity, and require minimal additional training. The biomarker targets would capture key pathological features with proven additive prognostic and clinical utility, such as the local inflammatory response and tumour microenvironment. The Glasgow Microenvironment Score (GMS), first described in 2014, combines assessment of peritumoural inflammation at the invasive margin with quantification of tumour stromal content. Using H&E sections, the Klintrup–Mäkinen (KM) grade is determined by qualitative morphological assessment of the peritumoural lymphocytic infiltrate at the invasive margin and tumour stroma percentage (TSP) calculated in a semi‐quantitative manner as a percentage of stroma within the visible field. The resulting three prognostic categories have direct clinical relevance: GMS 0 denotes a tumour with a dense inflammatory infiltrate/high KM grade at the invasive margin and improved survival; GMS 1 represents weak inflammatory response and low TSP associated with intermediate survival; and GMS 2 tumours are typified by a weak inflammatory response, high TSP, and inferior survival. The prognostic capacity of the GMS has been widely validated while its potential to guide chemotherapy has been demonstrated in a large phase 3 trial cohort. Here, we detail its journey from conception through validation to clinical translation and outline the future for this promising and practical biomarker.

## Introduction

Despite advances in early detection and management, colorectal cancer remains a leading cause of mortality worldwide. Over 1.9 million people were diagnosed with colorectal cancer in 2020, whereas 930,000 died from the disease in the same year [1]. Rates continue to rise in adults below the age of 50 and in countries transitioning to a higher income status [1], suggesting that CRC will remain a significant contributor to the global burden of disease for the foreseeable future.

Surgery supplemented with cytotoxic treatment including chemotherapy and radiotherapy is the keystone of curative treatment for patients with non‐metastatic CRC. Histopathological analysis of the resected specimen guides the use of adjuvant chemotherapy. High‐risk features of prognostic relevance beyond tumour size and nodal status include margin involvement [2, 3, 4], the presence of venous [5, 6, 7, 8, 9], lymphovascular [7, 9, 10, 11] or perineural invasion [12, 13, 14, 15], and satellite tumour deposits [16]. Recording of such features is now routine in core pathology reporting datasets [17].

Although standardised reporting provides quality assurance, higher rates of identification of adverse features and subsequent use of risk‐reducing adjuvant therapy have not significantly improved CRC survival in recent years. Patients with stage II (node negative) disease have 5‐year net survival of 84% falling to 65% in patients with stage III (node positive) disease [18]. Variation in survival among patients with similar stage disease is well recognised, particularly in stage II disease, highlighting the urgent need for more accurate and timely methods of identifying and managing patients at highest risk of poor outcome.

The development of a biomarker that increases the prognostic yield from routine histopathological analysis and uses methods that can be efficiently but reliably applied in clinical practice with direct therapeutic relevance represents a critical step in the journey to improved CRC outcomes. As part of this endeavour, several prognostic markers have been examined with multiple evaluations of the immune and inflammatory responses. In 1986, Jass highlighted the stage‐independent positive prognostic association of a strong lymphocytic infiltrate at the invasive margin of the tumour [19]. Two decades later, the Immunoscore was described by Galon *et al* [20], quantifying the density of CD3^+^ and CD45RO^+^ lymphocytes at the invasive margin and the tumour core. Although prognostication was enhanced beyond that of current TNM (tumour, node, metastasis) staging, the requirement for extra immunohistochemistry (IHC) sections and proprietary software for analysis have confined the Immunoscore to the realms of research rather than clinical practice.

Immediately prior to the initial description of the Immunoscore, Klintrup *et al* adapted the methodology of Jass to facilitate peritumoural lymphocytic infiltrate on routine haematoxylin and eosin (H&E) sections [21]. The qualitative morphological assessment of peritumoural inflammatory response ultimately resulted in the derivation of low versus high groups which were reproducible and independently prognostic of 5‐year survival [21]. Although the antitumour immune response was recognised as a key influence on outcome, Hanahan and Weinberg's seminal Hallmarks series of publications [22, 23, 24] highlighted the tumour microenvironment (TME) as an essential contributor to cancer development. The stromal component of the TME exerts a dominant influence on development, progression, and metastasis of CRC, with cancer‐associated fibroblasts a key mediator of this process.

Moves to assess the relative contribution of the stroma were spearheaded by Mesker *et al*, who first quantified this as the correlate of the carcinoma percentage (CP), defining a clinically relevant threshold of 50% [25]. A high tumour stroma percentage (TSP) was an independent adverse prognostic factor for both disease‐free survival (DFS) and overall survival (OS) [25]. This has subsequently been extensively validated in CRC [26, 27, 28, 29, 30], with therapeutic relevance in identifying patients with high‐risk stage II disease who may benefit from chemotherapy [31].

The advent of widespread ‐omics technology heralded an era of molecular‐based CRC classification systems. The original five subtypes based on DNA microsatellite instability (MSI), CpG island methylator phenotype status, presence of *KRAS* or *BRAF* mutations, and origin (serrated/adenomatous) described by Jass [32] were later subsumed by the international CRC Subtyping Consortium who analysed gene expression data from 4,151 patients [33]. They reported four consensus molecular subtypes (CMS) in 2015 comprising immune, metabolic, canonical, and mesenchymal groups. These were independently prognostic and showed promise in predicting response to chemo‐, immuno‐, and targeted therapies, yet clinical use of CMS has been limited. The cost and time associated with gene expression‐based subtyping, as well as the significant proportion (13%) of samples in the original study deemed unclassifiable have largely restricted its translation.

Transcriptomic subtyping followed, using patient‐derived xenografts to overcome contamination from stroma‐derived signatures. Five CRC intrinsic subtypes (CRIS) were defined, with further sub‐classification into two groups based on shared characteristics [34]. Similar to the CMS, the CRIS subtypes were independently prognostic. The CRIS subtypes represent a more robust method for segregating disease than the CMS due to utilisation of epithelial gene expression not confounded by stromal content [35]. However, clinical translation has again not been realised, largely due to the use of techniques which are not routine within existing diagnostic pathology resources.

It is clear that, while sophisticated molecular subtyping classifications have advanced our understanding of the heterogeneity of CRC, clinical translation is unlikely. Combinatorial scores assessing both inflammatory and stromal components, which employ routine H&E‐based methods are more readily applicable and cost‐effective solutions to the problem of identifying those at highest risk of poor outcome from CRC. The Glasgow Microenvironment Score (GMS) combines assessment of peritumoural inflammation at the invasive margin using the Klintrup–Mäkinen (KM) method with quantification of stromal content within the tumour, producing three prognostic categories with direct clinical relevance [36]. Here, we detail its journey from conception through validation to clinical translation and outline the future for this promising and practical biomarker.

## The conception of a prognostic biomarker

By 2015, it was well recognised that cancer cells were only one component of the TME, each with significant prognostic value. Indeed, the triumvirate of tumour cells, tumour‐associated stroma, and peritumoural inflammatory response were established as dynamic partners contributing to the processes facilitating invasion and acquisition of metastatic capacity. Scores focusing on single characteristics such as Immunoscore had been described over a decade earlier, with limited real‐world translation.

It was in this context that Park *et al* first described the GMS [36]. Combining the KM grade [21] and the TSP [25], both stage‐independent prognostic scores in patients with primary operable cancer, the GMS was examined in a single centre cohort of 307 patients undergoing elective, curative intent resection of stage I–III colorectal cancer [36]. Follow‐up was mature with a median of 126 months, during which 95 patients (31%) died from CRC.

**Figure 1:**
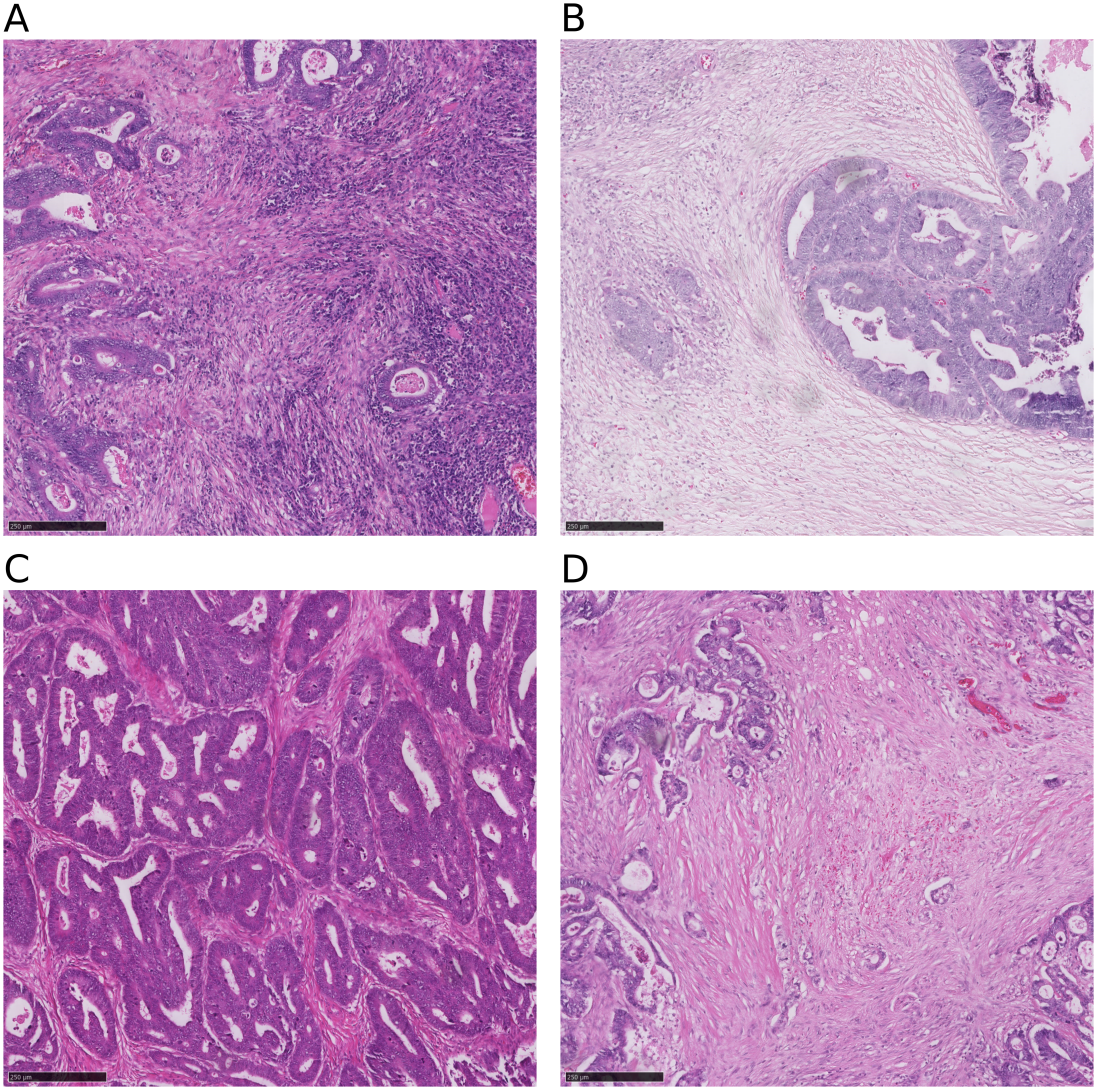
H&E‐stained sections assessed for tumour inflammatory cell infiltrate and TSP. (A) High KM grade with florid cup‐like infiltrate at the invasive edge with destruction of cancer cell islands. (B) Low KM grade displaying no increase in inflammatory cells at the invasive margin. (C) Low TSP with less than 10% tumour stroma. (D) High TSP with approximately 80% tumour stroma.

Utilising routine H&E‐stained sections of the deepest point of tumour invasion, the density of inflammatory cells at the invasive margin was graded using a four‐point scale and classified as low grade or high grade [21]. The former was defined as no increase or a mild or patchy increase in inflammatory cells, while the latter represented a prominent inflammatory reaction forming a band or a florid cup‐like infiltrate at the invasive margin. The proportion of stroma was calculated as a percentage of the visible field, excluding areas of mucin deposition or necrosis. Tumours were subsequently graded as low TSP (≤50%) or high TSP (>50%) [25] (Figure 1).

**Figure 2:**
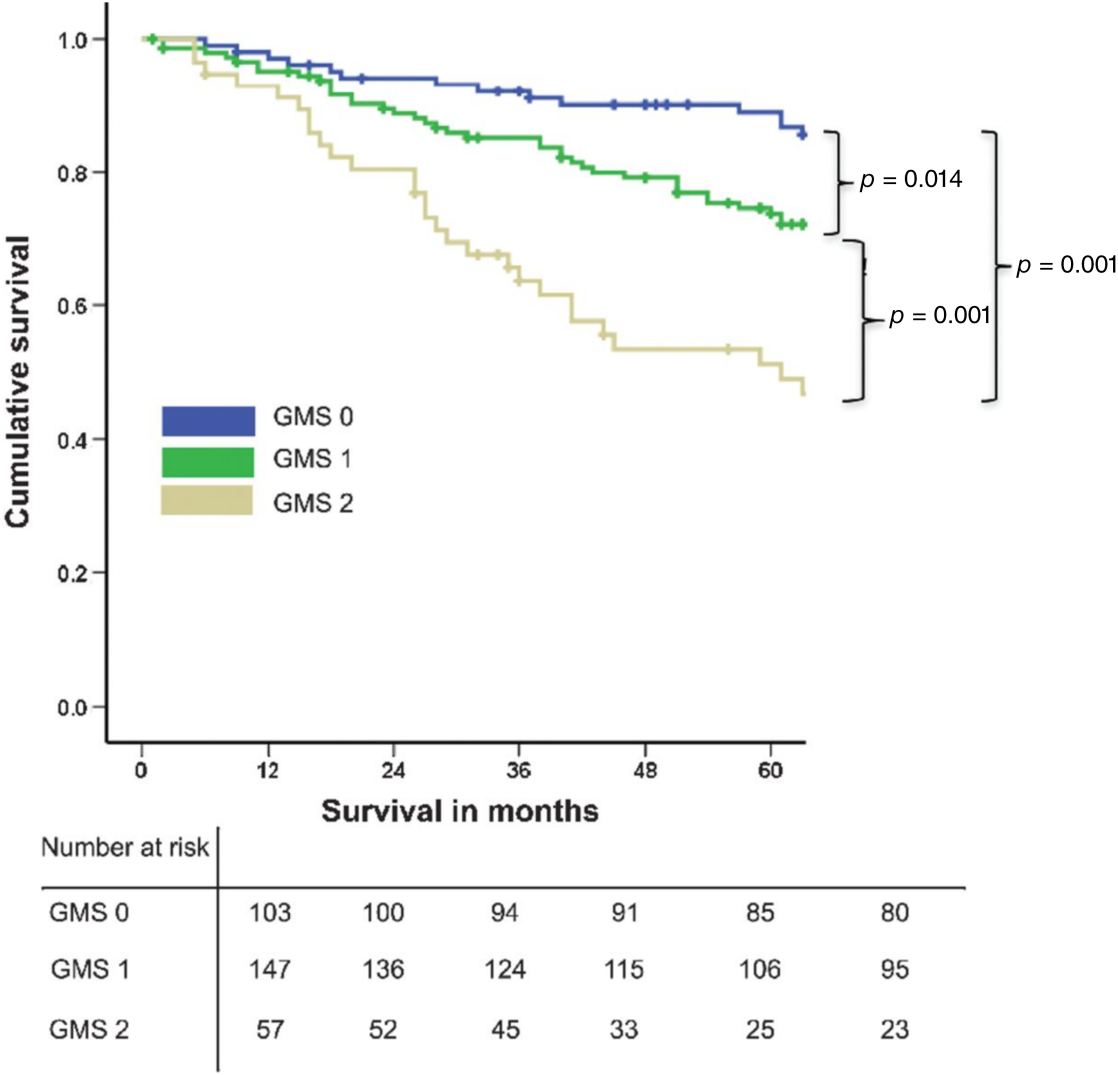
Kaplan–Meier plot depicting the relationship between the GMS and cancer‐specific survival in patients undergoing elective, potentially curative resection of colorectal cancer. Reproduced from Figure 2, Park *et al* [36] with permission.

On multivariate analysis, weak KM grade was associated with a hazard ratio (HR) of 2.00 (95% confidence interval [CI] 1.10–3.63, *p* = 0.022) while high TSP was similarly prognostic (HR 2.14, 95% CI 1.28–3.57, *p* = 0.004). Conversely, low TSP was associated with a 5‐year survival of 80%, while strong KM grade conferred a prognosis of 90% at 5 years. The GMS was subsequently derived as shown in Table 1. Following the observation that univariate HRs and 95% CIs for weak KM grade and high TSP overlapped, the presence of either characteristic was assigned a score of 1. Three groups were defined, with strong KM grade equalling 0, weak KM but low TSP corresponding to 1, and weak KM and high TSP totalling 2. The GMS categories offered more accurate stratification of 5‐year survival, with GMS 0 associated with good prognosis (5‐year survival 89%) and GMS 2 associated with a significantly worse prognosis [5‐year survival 51%, HR 4.08 (2.29–7.27)] (Table 1 and Figure 2).

**Table 1:** Tumour microenvironment characteristics and CSS in 307 patients undergoing elective, curative intent colorectal cancer resection (adapted from Park *et al* [36] with permission)

Characteristic	*N*	5‐Year CSS (%; SE)	Univariate HR (95% CI)	*p*
KM grade				
Strong	103	90 (3)	–	–
Weak	204	68 (3)	–	–
TSP				
Low	231	80 (3)	–	–
High	76	62 (6)	–	–
Combined KM grade/TSP				
0 (KM strong/low TSP)	84	89 (4)	1	–
1 (KM strong/high TSP)	19	89 (7)	1.23 (0.41–3.71)	0.715
1 (KM weak/low TSP)	147	75 (4)	2.00 (1.12–3.58)	0.020
2 (KM weak/high TSP)	57	51 (7)	4.25 (2.28–7.92)	<0.001
GMS				
0 (KM strong)	103	89 (3)	1	–
1 (KM weak/low TSP)	147	75 (4)	1.92 (1.13–3.28)	0.017
2 (KM weak/high TSP)	57	51 (7)	4.08 (2.29–7.27)	<0.001

The GMS was further explored in this cohort in relation to adverse pathological characteristics. Survival was significantly stratified using the GMS regardless of lymph node involvement, receipt of adjuvant chemotherapy, mismatch repair (MMR) status, or presence of venous invasion. The enhanced risk stratification offered by the GMS was most starkly demonstrated when comparing 5‐year survival of patients with lymph node‐negative disease with a GMS of 2 (69%) to patients with lymph node‐positive disease and a GMS of 0 (81%). Critically, reliability was good with inter‐rater correlation coefficient over 0.8 for both assessment of KM grade and TSP. However, the cohort was relatively small and limited to a single centre, with only MMR status as a molecular prognostic marker available. While the GMS displayed promise as a potential prognostic marker, exploring its relationships with survival in a larger, ideally external cohort was pivotal to progressing along the path of biomarker development.

## Strengthening the evidence: the first step towards GMS validation

Determining whether the GMS was replicable in an independent cohort was the next key step in exploring its validity as a prognostic marker. This was undertaken in a cohort of 862 patients with stage I–III CRC, incorporating 231 patients from the centre in which it was originally defined (Glasgow Royal Infirmary), supplemented by 631 patients from other centres in Glasgow (Western Infirmary, Gartnavel General, and Stobhill Hospitals) [37]. The same methodology as described above was used to determine the GMS: 300 patients were classified as GMS 0 (35%), 424 as GMS 1 (49%), and 138 as GMS 2 (16%). Again, follow‐up was mature at a median of 7 years, with 554 deaths and 271 recurrence events.

**Figure 3:**
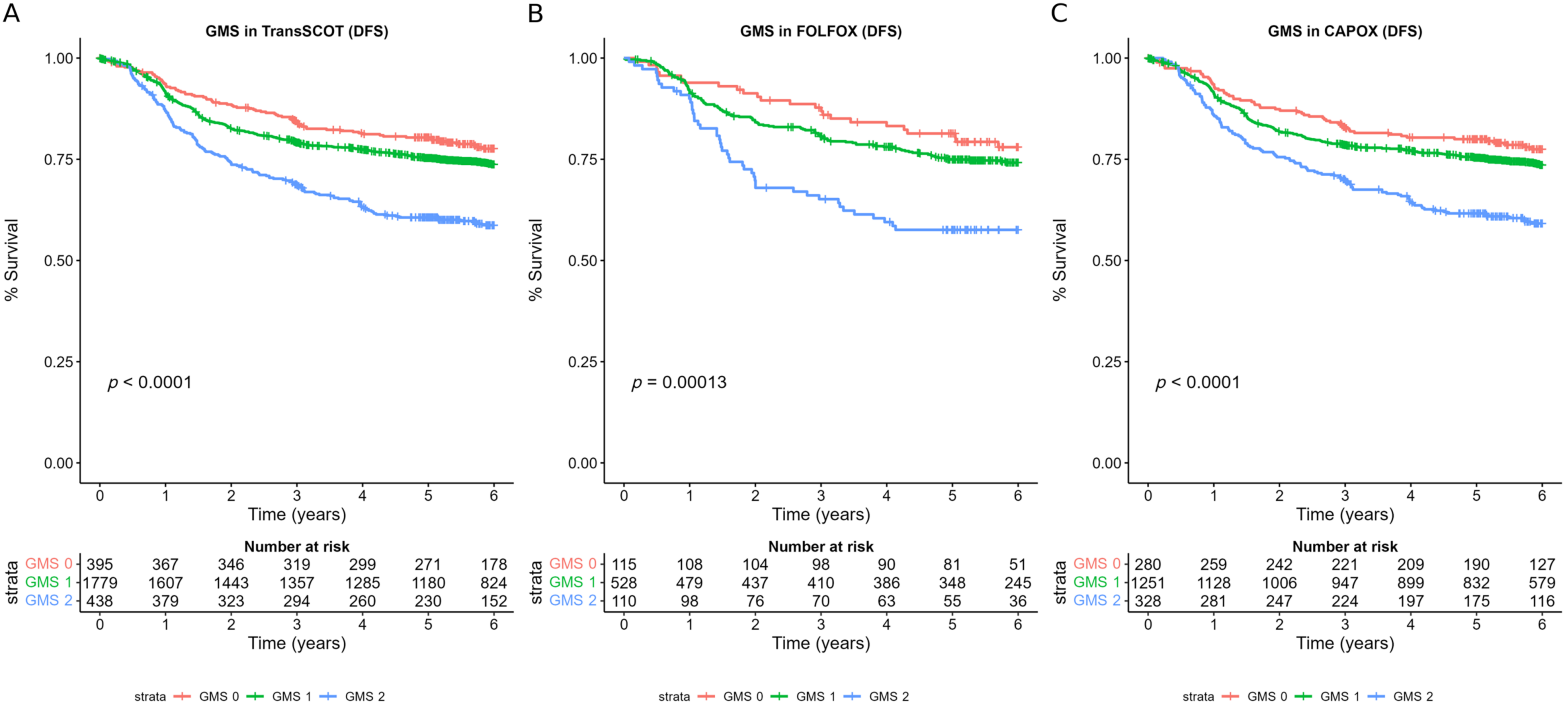
GMS‐stratified DFS among the TransSCOT cohort. The relationship between the GMS and DFS in (A) the TransSCOT cohort, (B) those treated with FOLFOX, and (C) those treated with CAPOX.

Using DFS at 5 years as the end point, GMS stratified survival in the whole cohort for at 71%, 58%, and 46% for GMS 0, 1, and 2, respectively, with a HR of 1.50 (95% CI 1.16–1.93, *p* = 0.002) for GMS 0 versus GMS 2 [37] (Figure 3). Moreover, GMS remained significantly associated with 5‐year DFS on multivariate analysis, independent of age (*p* < 0.001), T‐stage (*p* = 0.003), N‐stage (*p* < 0.001), and systemic inflammation as represented by the modified Glasgow Prognostic Score [38] (*p* < 0.001) [37]. Subgroup analysis highlighted that, although the GMS significantly stratified DFS in patients with colon cancer (*n* = 650, 75%), this was not the case in patients with rectal cancer (*n* = 212, 25%) [37].

Similar relationships were evident when assessing the GMS with the end point of recurrence‐free survival (RFS) at 5 years [37]. RFS was significantly stratified at 83%, 70%, and 51% for GMS 0, 1, and 2, respectively, with a HR of 3.09 (95% CI 2.19–4.36, *p* < 0.001) for GMS 0 versus GMS 2. However, in contrast to DFS, GMS stratified RFS in both colon and rectal cancers on subgroup analysis: in colon cancer, GMS 0, 1, and 2 had 5‐year RFS of 84%, 69%, and 51%, respectively (GMS 0 versus GMS 2: HR 3.15, 95% CI 2.08–4.77, *p* < 0.001) and in rectal cancer, 5‐year RFS for GMS 0, 1, and 2 was 80%, 72%, and 51%, respectively (GMS 0 versus GMS 2: HR 2.95, 95% CI 1.58–5.48, *p* = 0.001). Pattern of recurrence also varied by GMS category, with increasing recurrence risk as GMS increased (GMS 0 – 15%, GMS 1 – 26%, GMS 2 – 41%, *p* < 0.001). This was mainly driven by distant recurrence, but notably patients with GMS 2 were also more likely to develop local recurrence compared with GMS 0 or 1 [37].

Potential confounders in this cohort were notable for the inclusion of patients with emergency presentation (*n* = 175, 20%) [37]. Increasing GMS was noted to be associated with emergency presentation (*p* = 0.002), as well as young age (*p* = 0.04) and higher T‐ and N‐stage (both *p* < 0.001) [37]. However, a subgroup analysis presenting the GMS in patients undergoing elective surgery only was not presented. Despite this, the case for GMS as a prognostic biomarker appeared compelling based on this multicentre study with a majority of patients from external centres. Assessing the reproducibility of the demonstrated relationships in a fully external, elective cohort represented the next critical milestone in the GMS biomarker journey.

## Ensuring external validity: applying the GMS in a randomised controlled trial cohort

To consolidate the evidence that the reliability and prognostic capacity of the GMS translated to independent external CRC patient cohorts, Alexander *et al* sought to validate it in a large clinical trial cohort while assessing potential interactions with chemotherapeutic regimens [37]. The recently reported SCOT trial [39], an international randomised phase 3 trial comparing 3 versus 6 months oxaliplatin‐based chemotherapy [CAPOX (capecitabine and oxaliplatin) or FOLFOX (bolus and infused fluorouracil with oxaliplatin)] in patients with high‐risk stage II and stage III colorectal cancer, was utilised. Of the 6,088 SCOT participants, the TransSCOT cohort [37] comprised 2,912 participants who had undergone curative intent resection in the UK between 2008 and 2013 with tissue available for assessment of TSP and KM grade and at least 3 years of follow‐up data.

In the TransSCOT cohort, the GMS significantly stratified 5‐year DFS with GMS 0, 1, and 2 of 69%, 63%, and 53%, respectively [37]. When grouped by disease site, the prognostic effect of the GMS was preserved in patients with colon cancer (GMS 0 versus GMS 2: HR 2.20, 95% CI 1.64–2.94, *p* < 0.001) but not in patients with rectal cancer (GMS 0 versus GMS 2: HR 1.74, 95% CI 0.85–3.57, *p* = 0.130) [37]. On multivariate analysis, the GMS remained prognostic independent of T‐stage and N‐stage (*p* < 0.001) and was associated with increasing T‐stage (*p* < 0.001), increasing N‐stage (*p* = 0.002), colonic disease (*p* = 0.021), and higher risk stage‐III disease (*p* < 0.001) [37].

Notably, the GMS demonstrated a significant association with adjuvant chemotherapy type (*p* = 0.01) but not chemotherapy duration (*p* = 0.64) [37]. Subsequently, this interaction was investigated by stratifying the GMS for chemotherapy regimen. The association of the GMS with DFS was more pronounced in patients receiving FOLFOX, conferring a 5‐year DFS of 88%, 62%, and 54% for GMS 0, 1, and 2, respectively (GMS 0 versus GMS 2: HR 3.50, 95% CI 1.88–6.50, *p* < 0.001), but diminished in patients receiving CAPOX, conferring a 5‐year DFS of 62%, 63%, and 53% for GMS 0, 1, and 2, respectively (GMS 0 versus GMS 2: HR 1.33, 95% CI 0.98–1.85, *p* = 0.07) [37].

When further stratified into each GMS subtype, the high immune subtype GMS 0 demonstrated a notable improvement in survival between patients receiving FOLFOX over CAPOX (HR 2.23, 95% CI 1.19–4.16, *p* < 0.001) [37]. No difference in survival between chemotherapy regimen was observed in the other GMS subgroups. Further notable findings in patients with GMS 0 were also demonstrated: in lower risk TNM stage III (T1–3/N1) patients, those with a GMS 0 benefited from FOLFOX over CAPOX (HR 2.93, 95% CI 1.01–8.45, *p* = 0.047), but this was not the case in patients with higher risk TNM stage III (T4 or N2) disease and GMS 0 (HR 1.69, 95% CI 0.68–4.20, *p =* 0.257) [37].

In essence, the work of Alexander *et al* [37] not only provided robust external validation of the GMS but also highlighted its potential as a combined prognostic and therapeutic biomarker in specific subsets of patients with CRC. The GMS may therefore represent the elusive, much needed precision medicine tool that has capacity to guide chemotherapeutic decision‐making.

## Advancing the use of GMS to the diagnostic pathway

Although a distinct advantage of the GMS is its derivation from routine H&E sections, it was as yet unclear whether it could be applied to diagnostic CRC biopsies and retain its clinical relevance. Preoperative treatment in the form of neoadjuvant chemoradiotherapy was well established in rectal cancer. Indeed, at the time Park *et al* [40] were investigating the feasibility and clinical utility of applying the GMS to preoperative biopsies, the FOxTROT trial published encouraging results on the use of neoadjuvant chemotherapy in locally advanced colon cancer [41]. The validation of the GMS in the TransSCOT cohort provided rationale for using the GMS as a biomarker in adjuvant therapy. Clearly, a biomarker that reliably highlighted patients at risk of poor outcome early in the diagnostic pathway would be desirable, such that risk‐reducing strategies including neoadjuvant therapy could be considered.

**Figure 4:**
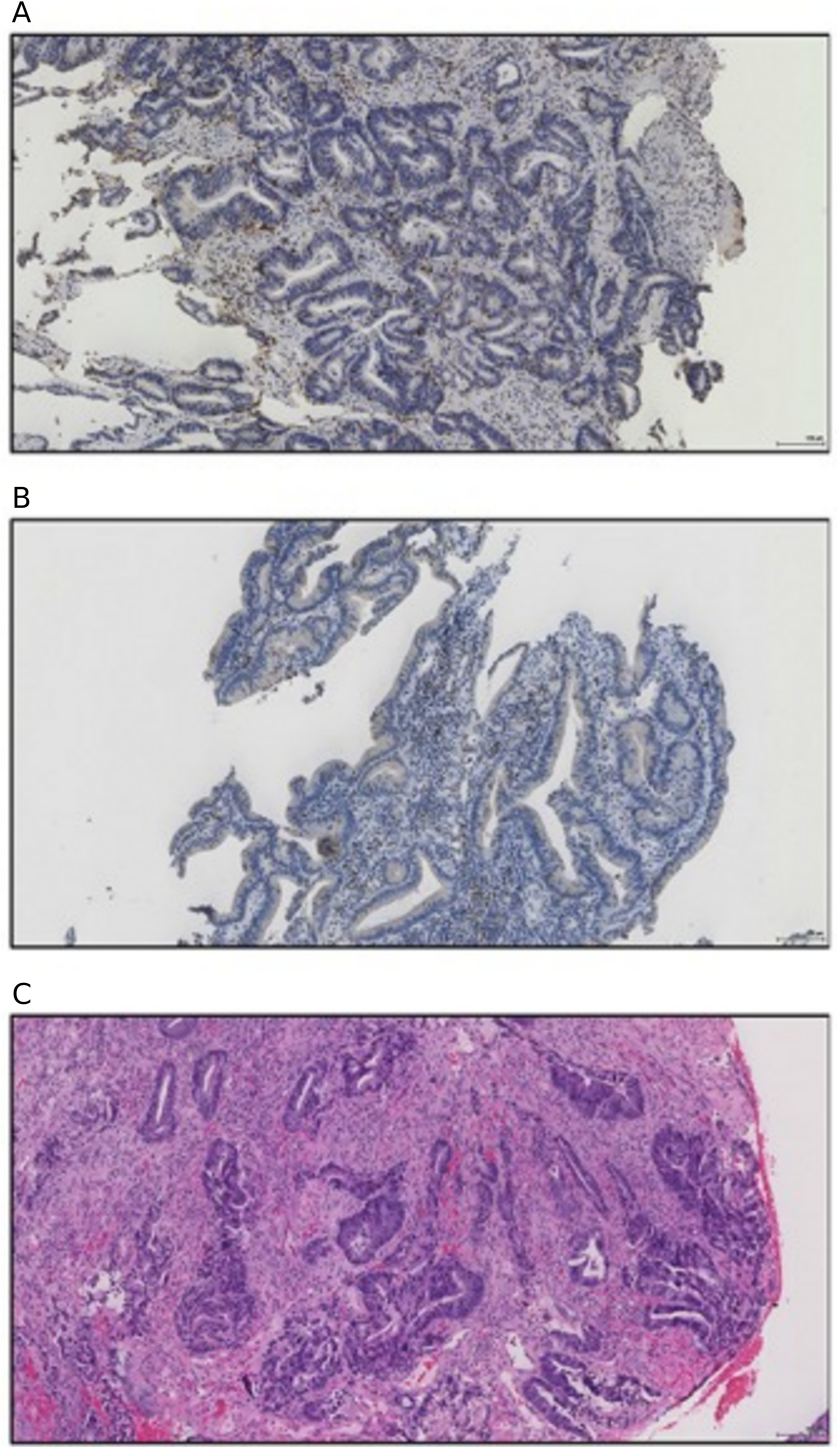
The biopsy‐derived Glasgow Microenvironment Score (bGMS). (A) Immunohistochemistry‐stained biopsy specimen showing high intra‐epithelial CD3^+^ density (122 cells/HPF), (B) immunohistochemistry‐stained biopsy specimen showing low intra‐epithelial CD3^+^ density (9 cells/HPF), (C) biopsy specimen showing high tumour stroma percentage. Adapted from Figure 1, Park *et al* [40] in accordance with the Creative Commons license (http://creativecommons.org/licenses/by/4.0/).

The feasibility of using the GMS as a biomarker in the diagnostic phase was therefore explored in endoscopic biopsy specimens from patients who had subsequently undergone curative intent resection of stage I–III CRC [40]. The GMS methodology was adapted for biopsies since KM grade was not directly translatable. To facilitate immune cell count assessment of intra‐epithelial CD3^+^ T‐lymphocytes, IHC staining using primary CD3^+^ antibody following antigen retrieval was undertaken. Three representative 0.6 mm × 0.6 mm high‐power fields (HPFs) were then identified, and cell counts were manually assessed. Assessment of biopsy stromal percentage was also adapted from that described in oesophageal cancer biopsies [42], using regions where tumour cells were present circumferentially and grading the proportion of intra‐tumoural stroma as low (≤50%) or high (>50%). Examples of biopsy specimens assessed for GMS are shown in Figure 4 (Table 2).

**Table 2:** Biopsy GMS characteristics

	CD3^+^	TSP	Figure
bGMS 0	High	–	4A
bGMS 1	Low	Low	4B
bGMS 2	Low	High	4C

In total, 115 patients with matched biopsy and full resection specimens were assessed. High CD3^+^ density at the invasive margin, stroma, and intra‐epithelial compartments of the full resection specimen was significantly associated with a higher biopsy T‐lymphocyte count [40]. The optimal threshold for intra‐epithelial CD3^+^ density was derived using receiver operating characteristic curves with a threshold of 25 cells/HPF. Biopsy assessment of TSP was also associated with TSP on full section (*p* = 0.001) [40]. However, the association was less robust than that between biopsy and full section CD3^+^ counts; 60% of biopsy samples classified as high TSP had corresponding full section TSP that were in fact low. Despite this, multivariable analysis confirmed biopsy CD3^+^ density (HR 0.23, 95% CI 0.09–0.57, *p =* 0.002) and biopsy TSP (HR 2.23, 95% CI 1.09–4.58, *p* = 0.029) to be associated with cancer‐specific survival (CSS), independent of TNM stage, venous invasion, and margin involvement [40]. The relationship between biopsy‐derived GMS (bGMS) and CSS was then explored. Akin to the original, full‐section GMS, bGMS stratified patients' 5‐year CSS into three prognostic groups: bGMS 0 – 92% (*n* = 53), bGMS 1 – 76% (*n* = 34), and bGMS 3 – 51% (*n =* 28), respectively (*p* < 0.001) [40].

Reproducibility was excellent with an inter‐rater reliability for assessment of biopsy intra‐epithelial CD3^+^ density of 0.866 and TSP of 0.743, both *p* < 0.001 [40]. Nonetheless, technical factors related to biopsy specimen quality resulted in incorrect classification of 23 patients (20%). The availability of sufficient tissue to assess three HPFs for intra‐epithelial CD3^+^ lymphocytes was limited to 91 patients (79%), with the remainder evenly split between those with 2 HPFs and 1 HPF available [40]. Recognising such factors restrict the diagnostic capacity of biopsy specimens in clinical practice, the feasibility and reliability of biopsy‐based GMS derivation was established, paving the way for its clinical translation.

## Expanding the evidence base

From conception through iterative validation to derivation in biopsies, the GMS was demonstrated to be both versatile and reliable. Attempts have been made by external groups to combine TME characteristics for prognostic purposes. Using similar methodology but post‐dating the derivation of the GMS, Hynes *et al* reported a combined fibro‐inflammatory score incorporating TSP and peritumoural inflammation [26]. Scored from 0 to 3, combined scores of 2 or more were associated with poorer CSS (HR 2.44, 95% CI 1.56–3.81, *p* < 0.05) among 445 patients with TNM stage II/III colon cancer drawn from a national cancer registry [26].

Subsequent studies characterising the GMS in relation to contemporary prognostic factors have added further context to its clinical relevance (Table 3). To address whether the prognostic value of the GMS was maintained with regard to MMR status, Alexander *et al* utilised a cohort of 783 TNM I–III colorectal cancers, for whom MMR status was available in 771 [43]. An increasing GMS was associated with MMR deficiency (*p* = 0.02). For CSS, the GMS stratified both MMR‐proficient and MMR‐deficient disease with 5‐year CSS of 93%, 80%, and 65%, and 95%, 75%, and 59%, respectively (MMR proficient: GMS 0 versus GMS 2: HR 3.21, 95% CI 1.76–5.84, *p* < 0.001; MMR deficient: GMS 0 versus GMS 2: HR 6.72, 95% CI 1.53–29.58, *p* = 0.02) [43]. For OS, the GMS also stratified MMR‐proficient and MMR‐deficient disease with 5‐year OS of 75%, 63%, and 49%, and 68%, 60%, and 38%, respectively (MMR proficient: GMS 0 versus GMS 2: HR 1.84, 95% CI 1.26–2.70, *p* = 0.007; MMR deficient: GMS 0 versus GMS 2: HR 2.23, 95% CI 1.13–4.41, *p* = 0.02) [43]. Interestingly, MMR status was not associated with recurrence in this cohort on regression analysis, while higher GMS was independently related to recurrence, whether combined local and systemic or distant [43]. It is clear, therefore, that the GMS appears to add value rather than act as a proxy for existing prognostic markers such as MMR status.

**Table 3:** The prognostic value of the GMS and other prognostic markers in colorectal cancer

Prognostic marker, HR (95% CI)	GMS, HR (95% CI)	Prognostic status	Cohort	End point	Study
Differentiation grade					
HR not given, *p* = 0.46	1.24 (1.07–1.43), *p* = 0.004	Independent	*n* = 862	RFS	[37]
TNM stage					
1.73 (1.07–2.80), *p* = 0.025	1.93 (1.36–2.73), *p* < 0.001	Independent	*n* = 307	CSS	[36]
Nodal involvement					
1.73 (1.48–2.03), *p* < 0.001	1.28 (1.12–1.47), *p* < 0.001	Independent	*n* = 2,912	DFS	[37]
Peritoneal involvement					
Chi‐squared analysis, *p* = 0.004	–	Not assessed	*n* = 307	–	[36]
Margin involvement					
Chi‐squared analysis, *p* = 0.003	–	Not assessed	*n* = 307	–	[36]
Venous invasion					
2.37 (1.42–3.94), *p* = 0.01	1.93 (1.36–2.73), *p* < 0.001	Independent	*n* = 307	CSS	[36]
MMR status					
HR/CI not given, *p* = 0.08	1.54 (1.19–2.00), *p* < 0.001	Independent	*n* = 771	CSS, OS	[39]
Tumour budding					
4.03 (2.50–6.52), *p* < 0.001	1.54 (1.15–2.07), *p* = 0.004	Independent	*n* = 303	CSS	[40]
*KRAS* status					
1.16 (0.84–1.59), *p* = 0.37	1.24 (1.07–1.43), *p* = 0.004	Independent	*n* = 212	RFS	[37]
*BRAF* status					
1.03 (0.66–1.59), *p* = 0.90	1.24 (1.07–1.43), *p* = 0.004	Independent	*n* = 212	RFS	[37]

**Figure 5:**
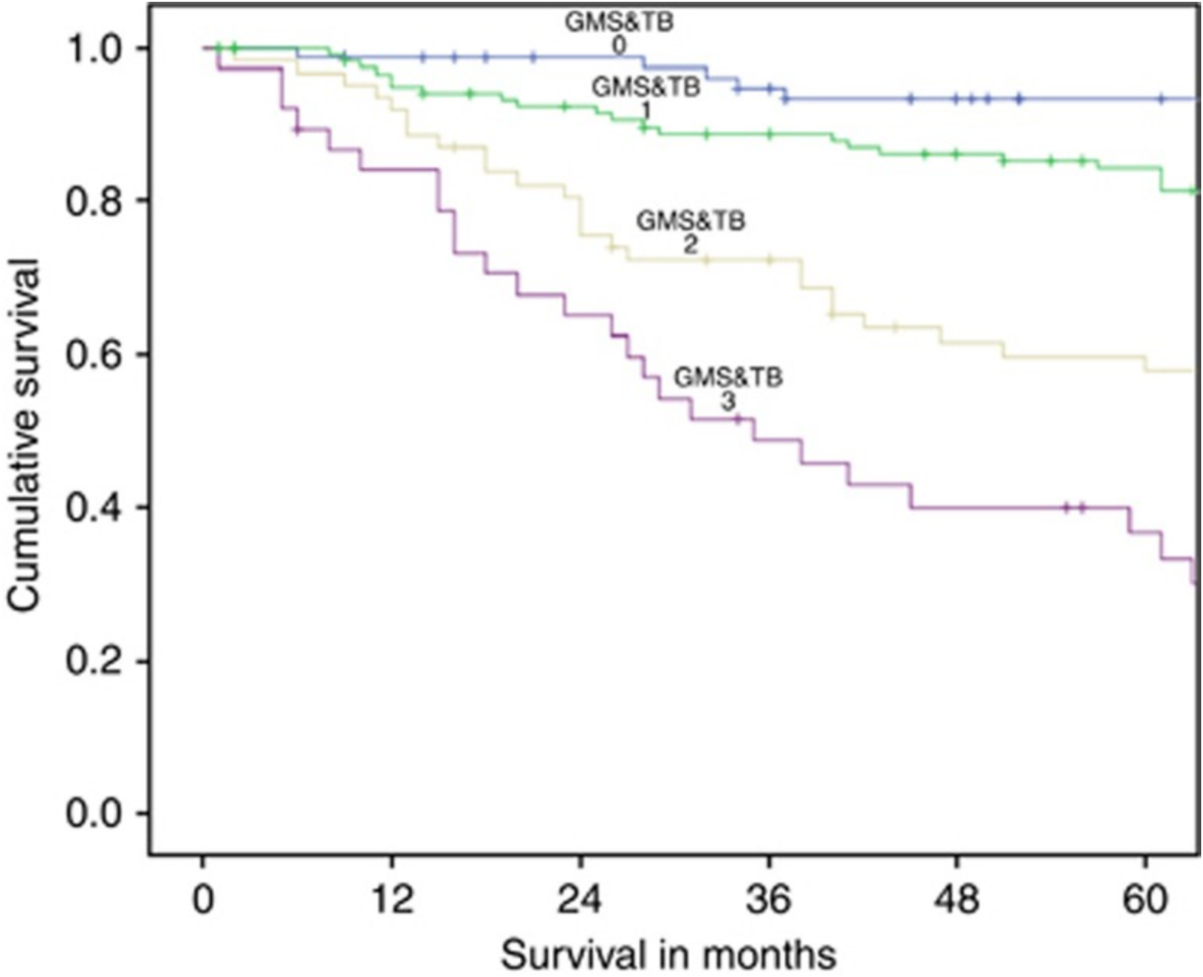
The relationship between combined Glasgow Microenvironment Score–tumour budding and cancer‐specific survival in patients with primary operable colorectal cancer (*p* < 0.001). Reproduced from Figure 4, van Wyk *et al* 2016 [44] in accordance with the Creative Commons license (https://creativecommons.org/licenses/by‐nc‐sa/4.0/).

Tumour budding, a marker of aggressive tumour behaviour and the process of epithelial–mesenchymal transition (EMT), has recently been incorporated as a component of the Royal College of Pathologists Colorectal Cancer Reporting guidelines [17]. van Wyk *et al* assessed the interaction between tumour budding and the TME as characterised by the GMS [44]. Notably, tumour budding was significantly associated with a higher proportion of tumour stroma and a weaker inflammatory cell infiltrate. Indeed, high‐grade budding was associated with increasing GMS and independently prognostic of CSS alongside age, TNM stage, venous invasion, and GMS. When combined, GMS and tumour budding effectively stratified survival in patients with primary operable colorectal cancer (HR 2.16, 95% CI 1.65–2.82, *p* < 0.001) [44] (Figure 5).

More recently, the association between the GMS and specific markers of EMT was explored [45]. Among E‐cadherin, β‐catenin, Fascin, Snail, and Zeb1, nuclear β‐catenin was the only marker associated with the GMS as a whole (*p* = 0.03). High nuclear β‐catenin expression was notably associated with higher GMS (68% GMS 2 versus 47% GMS 0). GMS 0 tumours displayed the lowest levels of Fascin expression, a finding that is consistent with its recognised role in cell motility and migration.

The GMS has also been explored in relation to immune checkpoint proteins in patients with primary operable colorectal cancer [46]. High TIM‐3 expression on immune cells located within the stroma was associated with low GMS (*p* < 0.001). Similarly, PD‐1 expression within immune cells located in the stroma was associated with low GMS (*p* < 0.001) [46]. Such studies have reinforced the original derivation of the GMS groupings, confirming expected associations with high immune GMS 0 subtype and demonstrating its consistency in relation to novel pathological factors. In future, GMS assessment in patients receiving immunotherapy may clarify whether it is associated with therapeutic response in a manner similar to that seen with adjuvant chemotherapy in the TransSCOT cohort.

## Translation to clinical practice: ensuring robust reliability

The key barriers to the adoption of histopathological subtyping methods such as the GMS in routine diagnostic procedures are the lack of standardised scoring criteria and concerns over the inter‐observer reliability of assessment. However, significant strides have been made to address these issues for the TSP component of the GMS.

The assessment criteria for the TSP have remained relatively consistent since its inception. Mesker *et al* originally determined that ×10 objective fields of the area with the lowest CP (highest TSP with tumour cells at all edges of the field) scored in 10% increments were sufficient to distinguish between high‐ and low‐risk patients for both DFS and OS [25]. This scoring criterion was subsequently validated for DFS and OS in stage II/III patients with CRC in the VICTOR trial with high inter‐observer agreement (Cohen's Kappa = 0.89) [31].

This criterion was further refined by Park *et al* in 2014, where they demonstrated that a single ×10 objective field per H&E section scored to the nearest 5% to improve granularity was sufficient to determine the prognostic effect of the tumour's stromal component while maintaining high inter‐observer reliability. The intraclass correlation coefficient (ICCC) of the TSP was 0.783 and Cohen's Kappa of the TSP group was 0.813 between two observers [28]. Subsequently, the TSP has been validated both for prognostic significance and inter‐observer reliability in a multitude of studies demonstrating consistently strong agreement, with reported Kappa values ranging from 0.6 to 0.97 [47].

Furthermore, studies comparing the TSP to tumour budding, which is regularly assessed in clinical practice, have demonstrated that TSP assessment shows comparable and, in some cases, greater inter‐observer reliability [48, 49, 50]. In 2019, the Uniform Noting for International Application of the Tumour‐Stroma Ratio as an Easy Diagnostic Tool (UNITED) group developed an e‐learning tool consisting of instructional material and sets of reference slides to aid pathologists in reliably and reproducibly scoring the TSP according to the established methodology. Following completion of the instructional material, pathologists scored an initial reference set of 40 slides and, if a Kappa ≥ 0.7 was achieved, scored a further 40 reference slides which were repeated after 2 months to establish inter‐ and intra‐observer variability [51]. The results demonstrated that, among 31 participants (23 pathologists and 8 residents) completing the study, there was a significant improvement in agreement from the training to the test set [training set median Kappa 0.72 (range = 0.21–0.90) and test set median Kappa 0.77 (range = 0.51–0.97)], with no decrement after a 2‐month washout period, *p* = 0.74 [52].

Although a number of studies have investigated the KM grade for its effect on prognosis, relatively few have examined its inter‐observer reliability. The original study establishing the KM criteria investigated the measures of inter‐observer reliability between six observers (three pathologists and three residents) and the intra‐observer reliability of a single pathologist. These were conducted using the binary high versus low scoring used in the GMS rather than the original four‐point system and confirmed good inter‐observer [mean Kappa = 0.672 (range = 0.504–0.794)] and excellent intra‐observer (Kappa = 0.794) agreement. However, no information was provided on the length of the washout period between assessments [21].

In 2009, Roxburgh *et al* assessed the KM criteria for prognosis in node‐negative disease and reported good inter‐observer agreement using the four‐point scale, with an ICCC of 0.81 between two observers in a 100‐patient cohort [53]. Using a modified version of the criteria, restricted to lymphoid infiltrate only with no other inflammatory cell types included, Hynes *et al* found that inter‐observer agreement for the binary score was poor to fair among four pathologists (Kappa range = 0.05–0.48). However, intra‐observer variability after a 6‐week washout period with a single pathologist was excellent (Kappa = 0.79) [26]. It is plausible that the lower inter‐observer reliability in this study results from the need to identify an individual immune cell type rather than assessing the total inflammatory response. Discerning specific inflammatory cell types has previously resulted in notable variability in terms of agreement [54].

While omics‐based subtyping provides a more granular insight into the mutational status of the tumour and, indeed, may be more advantageous for certain clinical applications, such as identifying druggable mutations on a per patient basis, its clinical use remains limited by cost and resource availability. Moreover, omics‐based subtyping is associated with a degree of attrition, with 13% of patients unassigned by the CMS [33], whereas the GMS assigns all patients to a group contingent on the diagnostic section having an identifiable invasive margin.

Histopathological approaches to recapitulate features of omics‐based subtyping methods have been identified with some success. Trinh *et al* demonstrated that an IHC‐based five‐protein panel (CDX2, FRMD6, HTR2B, ZED1, and KER) coupled with MSI distinguished the CMS with 87% concordance. It was, however, unsuccessful in separating CMS2 and three tumours and would require the validation of each of these novel markers in the diagnostic setting [55].

In their article describing translation of the phenotypic subtypes of CRC to routine diagnosis, Roseweir *et al* identified that phenotypic components of the GMS translate some of the biological signatures comprising the CMS to histopathological assessment, notably the prominent inflammatory infiltrate of CMS1 in GMS0 and the dense stromal reaction associated with CMS4 in GMS2 [56]. Indeed, the phenotypic subtypes expand upon the GMS by utilising Ki67 IHC to further stratify GMS1. Although Ki67 is not validated for clinical use in CRC, it is validated for molecular subtyping of breast cancer and could therefore represent an evolution of the GMS in clinical practice [57].

## Future directions – computational pathology

Standardised assessment protocols and multicentre validation studies have bolstered the process of clinical translation of the GMS. Recent developments in the application of deep learning to computational pathology could provide a further avenue for the GMS to enter routine clinical use.

One of the first challenges in applying deep learning in pathology research was accurate pixel‐wise segmentation of histopathology images, driving the development of models specifically for this task [58]. Quantification of the TSP is therefore a logical next step following classification of the tissue and a number of studies have examined this in terms of segmentation accuracy, prognosis, and agreement with pathologist assessment.

In 2019, Geessink *et al* performed a comprehensive study on the use of deep learning to quantify the TSP in 154 patients with stage I–III rectal adenocarcinoma [59]. Using the same 1.8‐mm diameter field of view in which two pathologists assessed the TSP, they segmented the tissue into nine classes and found that the overall accuracy of the model was 94.6% compared with manual annotation. When comparing the raw percent scores determined by the model and the two pathologists, the ICCC values were 0.475 and 0.411 indicating moderate agreement, and with the TSP dichotomised at the standard 50% only fair agreement was found, with Cohen's Kappa = 0.239. However, the latter was notably improved when the model scores were dichotomised at the median value (65.47%), increasing the Kappa value to 0.521.

For survival, the deep learning model demonstrated better stratification for both disease‐specific survival and DFS. This discrepancy between model segmentation accuracy and agreement with manual assessment has been consistently highlighted by subsequent studies. Firmbach *et al* also noted a similar phenomenon for the dichotomised scores [60]. When comparing the two most senior observers from a pool of 10 to a deep learning model with an overall six‐class accuracy of 86.5%, the ICCC values were 0.750 and 0.689, and for the scores dichotomised at 50%, Kappa values were 0.400 and 0.333. However, with the scores dichotomised at 65%, agreement again notably improved (Kappa = 0.502 for both observers).

Further investigation revealed that the selection of the region of interest (ROI) in which the TSP was quantified contributed to variation in segmentation accuracy in the model and errors in assessment for both human observers and the model [60]. Smit *et al* further highlighted the importance of ROI selection by comparing a deep learning TSP model with manual and automatic ROI selection to pathologist assessment [61]. When applied to the same ROI as manual assessment, their model achieved an ICCC = 0.78 and a Spearman rank correlation = 0.88 compared to three observers. However, when an automated approach to ROI selection was applied, the correlation between the model and the three observers was notably lower (Spearman rank correlation = 0.75). While deep learning models will inevitably continue to improve and demonstrate greater segmentation accuracy, these studies highlight that, in clinical practice, pathologist intervention will likely always be required to ensure reliability.

**Figure 6:**
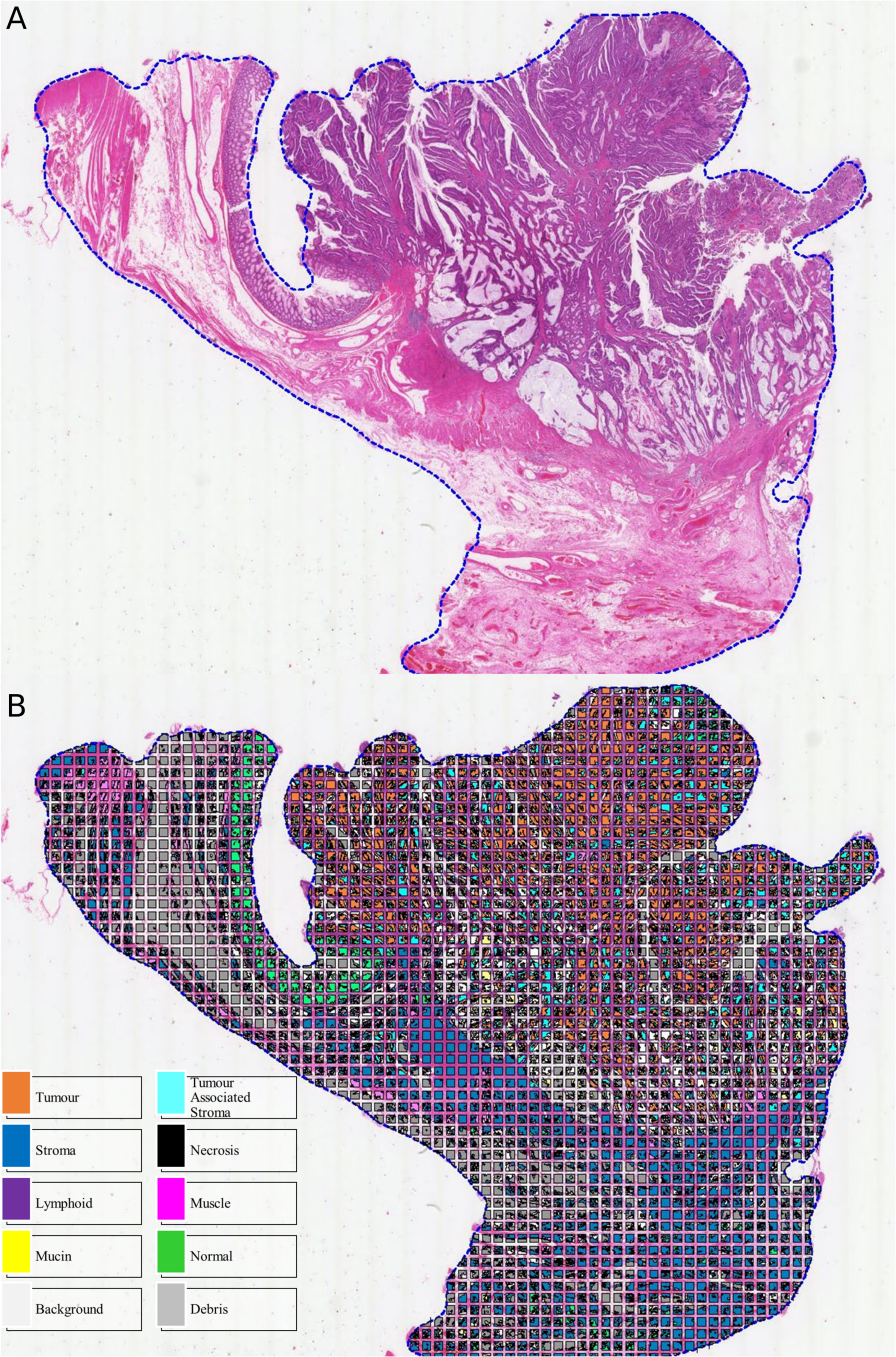
(A) Colorectal cancer H&E whole slide image, (B) following tissue segmentation by U‐Net convolutional neural network.

Translating the KM system to a computational pathology approach is a more challenging task due to the qualitative nature of the scoring criteria and the difficulty of automatically segmenting and classifying immune cell populations on H&E (Figure 6). A number of studies have shown that automated quantification of immune cell populations correlates with prognosis and demonstrates good agreement with pathologist assessment. Using the open source QuPath software [62], Väyrynen *et al* used a machine learning approach to quantify densities of lymphocytes, plasma cells, neutrophils, and eosinophils in H&E tissue microarray cores of colorectal cancer, with core‐level Spearman's Rho values of 0.95, 0.74, 0.71, and 0.84, respectively, against pathologist annotation. In 934 patients, they demonstrated that simple stromal densities of all immune cell subtypes significantly stratified patients for 10‐year CSS, and that assessing the spatial relationship with tumour cells provided more granular survival prediction [63].

Using deep learning‐based segmentation, Pai *et al* quantified a number of histopathological features on H&E whole slide images (WSI) in colorectal cancer, including tumour‐infiltrating lymphocyte (TIL) density, which showed excellent agreement compared to four observers, mean ICCC = 0.754, range = 0.564–0.867 [64]. Following a similar approach, Yang *et al* demonstrated that deep learning‐based quantification of stromal lymphocytes on H&E WSI significantly stratified 1,010 patients for survival and correlated with serial section CD3 on IHC WSI in a subset of 129 patients [65]. It is possible therefore that this approach could be utilised to automatically infer the KM grade, as Xu *et al* showed that there is sufficient variation in lymphocyte densities quantified by deep learning between KM grades at the invasive margin that also translate across patient cohorts [66].

However, this is not a direct translation, and it is likely that a combination of methods would be required to capture the granularity of the KM criteria. Given that the KM grade is essentially a qualitative assessment of the clustering patterns of immune cells at the invasive margin, the 2018 study by Saltz *et al* could provide a means by which to translate it to computational pathology. Their study utilised patch‐based segmentation to identify lymphocyte dense regions of H&E WSI, following which the spatial relationships between the patches were investigated for correlation with prognosis. Examining a variety of measures of local clustering patterns, they identified that different TIL spatial structures can have disease‐dependent associations with prognosis, and it is therefore possible that one or a combination of these in conjunction with simple density quantitation could provide a more faithful adaptation of the KM criteria [67].

## Conclusion

Since its original description in 2015, the journey of the GMS has exemplified the process of clinically relevant biomarker derivation in colorectal cancer. Supported by its systematic validation and ease of clinical translation, it is anticipated that the GMS will be incorporated into colorectal cancer reporting guidelines in a manner similar to contemporary prognostic markers such as tumour budding. Although this represents the end goal in biomarker development, the ultimate objective of translating the GMS to clinical practice lies in realising its potential to improve the accuracy of prognosis and the quality of therapeutic shared decision‐making between healthcare professionals and patients with colorectal cancer.

## Author contributions statement

JE and CR conceived and designed the review. KK, CB and JE developed the methodology. KK, CB and KP acquired the data. KK, CB, JE and JH analysed and interpreted the data (e.g. statistical analysis, biostatistics, computational analysis). JP, DM, JH, NM, CR and JE wrote, reviewed and/or revised the manuscript. JP, JH, NM, KP and CR provided administrative, technical or material support (i.e. reporting or organising data, constructing databases). DM, CR and JE supervised the study.
